# *In Vitro* Toxicity of Industrially Relevant Engineered Nanoparticles in Human Alveolar Epithelial Cells: Air–Liquid Interface *versus* Submerged Cultures

**DOI:** 10.3390/nano11123225

**Published:** 2021-11-27

**Authors:** Maria João Bessa, Fátima Brandão, Paul H. B. Fokkens, Daan L. A. C. Leseman, A. John F. Boere, Flemming R. Cassee, Apostolos Salmatonidis, Mar Viana, Adriana Vulpoi, Simion Simon, Eliseo Monfort, João Paulo Teixeira, Sónia Fraga

**Affiliations:** 1 Department of Environmental Health, National Institute of Health Dr. Ricardo Jorge, 4000-053 Porto, Portugal; mjbessa8@gmail.com (M.J.B.); fatimabrandao988@gmail.com (F.B.); sonia.fraga@insa.min-saude.pt (S.F.); 2 EPIUnit-Instituto de Saúde Pública, Universidade do Porto, 4050-091 Porto, Portugal; 3 Laboratório para a Investigação Integrativa e Translacional em Saúde Populacional (ITR), 4050-091 Porto, Portugal; 4 Instituto de Ciências Biomédicas Abel Salazar (ICBAS), Universidade do Porto, 4050-313 Porto, Portugal; 5 National Institute for Public Health and Environment (RIVM), 3721 Bilthoven, The Netherlands; paul.fokkens@rivm.nl (P.H.B.F.); daan.leseman@rivm.nl (D.L.A.C.L.); john.boere@rivm.nl (A.J.F.B.); flemming.cassee@rivm.nl (F.R.C.); 6 Institute for Risk Assessment Sciences (IRAS), Utrecht University, 3584 Utrecht, The Netherlands; 7 Institute of Environmental Assessment and Water Research, Spanish Research Council (IDAEA-CSIC), 08034 Barcelona, Spain; asalmatonidis@leitat.org (A.S.); mar.viana@idaea.csic.es (M.V.); 8 LEITAT Technological Center, C/de la Innovació 2, 08225 Terrassa, Spain; 9 Nanostructured Materials and Bio-Nano-Interfaces Center, Interdisciplinary Research Institute on Bio-Nano-Sciences, Babes-Bolyai University, 400271 Cluj-Napoca, Romania; adrianavulpoilazar@gmail.com (A.V.); simon49nmr@gmail.com (S.S.); 10 Institute of Ceramic Technology (ITC), Universitat Jaume I, 12006 Castellón, Spain; eliseo.monfort@itc.uji.es

**Keywords:** engineered nanoparticles, submerged cultures, air-liquid interface, *in vitro* cytotoxicity, DNA damage, genotoxicity

## Abstract

Diverse industries have already incorporated within their production processes engineered nanoparticles (ENP), increasing the potential risk of worker inhalation exposure. *In vitro* models have been widely used to investigate ENP toxicity. Air–liquid interface (ALI) cell cultures have been emerging as a valuable alternative to submerged cultures as they are more representative of the inhalation exposure to airborne nano-sized particles. We compared the *in vitro* toxicity of four ENP used as raw materials in the advanced ceramics sector in human alveolar epithelial-like cells cultured under submerged or ALI conditions. Submerged cultures were exposed to ENP liquid suspensions or to aerosolised ENP at ALI. Toxicity was assessed by determining LDH release, WST-1 metabolisation and DNA damage. Overall, cells were more sensitive to ENP cytotoxic effects when cultured and exposed under ALI. No significant cytotoxicity was observed after 24 h exposure to ENP liquid suspensions, although aerosolised ENP clearly affected cell viability and LDH release. In general, all ENP increased primary DNA damage regardless of the exposure mode, where an increase in DNA strand-breaks was only detected under submerged conditions. Our data show that at relevant occupational concentrations, the selected ENP exert mild toxicity to alveolar epithelial cells and exposure at ALI might be the most suitable choice when assessing ENP toxicity in respiratory models under realistic exposure conditions.

## 1. Introduction

Nanotechnology is one of the key technologies of the 21st century that is revolutionizing various fields of activity through the production and application of engineered nanomaterials (ENM). Carbon-based nanomaterials (NM), metal and metal oxide nanoparticles (NP) are amongst the most used ENM in the industrial sector, which are consequently being produced in high volumes [1,2]. Accordingly, nano-sized materials are considered an emerging risk for occupational safety and health [3,4] and there is an urgent need to clearly identify the adverse health effects associated with workplace exposure to NP. In this context, the ceramic sector is a relevant case of occupational exposure to NP. Indeed, a wide range of ENM are already being used as raw materials in advanced ceramics manufacture, including carbon-based NM (e.g., graphene, carbon nanotubes and carbon black) for their reinforcing ability or metal/metal oxide NP [e.g., aluminium oxide (Al_2_O_3_), antimony-tin oxide (ATO; Sb_2_O_3_•SnO_2_), cerium oxide (CeO_2_), chromium oxide (Cr_2_O_3_), silica (SiO_2_), tin oxide (SnO_2_), titanium oxide (TiO_2_) and zirconium oxide (ZrO_2_)] for ceramic coatings, as insulators, cutting tools and polishing agents [5]. In addition, nano-sized particles may be unintentionally released to workplace air during advanced, as well as traditional ceramics manufacturing processes such as machining, combustion/heating processes, thermal coating, etc. [5,6,7,8,9,10,11,12,13,14,15]. This has also been observed in other industrial sectors [16].

Inhalation is considered a major route of exposure to NP in occupational settings, though dermal contact and ingestion are also likely to occur [17,18]. Depending on physiological factors (breathing pattern and lung health status) [19] but also on NP physicochemical properties (size, shape, surface chemistry) [20], airborne NP will deposit at different locations along the respiratory tree, where they might or might not exert toxicity. The available studies on the toxicity of ENM show that cell injury may arise from particle–cell interactions, plasma membrane perturbation and/or loss of integrity, mitochondrial function disruption, elevation of reactive oxygen species (ROS) levels, among others [21,22,23].

A large proportion of the existing information on ENM-induced biological effects derives from *in vitro* studies using lung models. Human airway epithelial cell lines from the bronchial (e.g., 16HBE14o, BEAS-2B or Calu-3 cells) and alveolar regions (e.g., A549 cells) are the most used culture systems [24,25,26]. In this regard, human alveolar epithelial A549 cells are often employed for assessing the toxicity of nano-sized materials [27,28]. Indeed, alterations in alveolar epithelial cells integrity and function, which might occur from the presence of ENM in the lung tissue, are in the basis of severe pulmonary diseases [29].

Metal oxide NP are amongst the most widely investigated ENM for *in vitro* pulmonary toxicity. In this regard, Lanone et al. [30] evaluated the *in vitro* toxicity of 24 manufactured NP, including metal oxide NP, in both human alveolar epithelial (A549) and macrophage (THP-1) cells at 24 h after exposure. These authors found that chemical composition was an important determinant of ENM toxicity, while no correlation between cytotoxicity and NP equivalent spherical diameter or specific surface area was found. While copper oxide (CuO) and zinc oxide (ZnO) NP were the most cytotoxic NP, TiO_2_, Al_2_O_3_, CeO_2_ and ZrO_2_ NP induced moderated cytotoxicity. On the one hand, tungsten carbide (WC) NP did not cause any significant cytotoxicity. Importantly, A549 and THP-1 cells exhibited different sensitivity to the tested NP. In addition, Titma et al. [31] investigated the *in vitro* cytotoxicity of six metal oxide NP (antimony oxide (Sb_2_O_3_), manganese oxide (Mn_3_O_4_), TiO_2_, cobalt oxide (Co_3_O_4_), ZnO and CuO NP) in human alveolar epithelial (A549) but also in intestinal epithelial (Caco-2) cells. In both cell models, no toxic effects were observed in cells exposed for 24 h to Sb_2_O_3_, Mn_3_O_4_ and TiO_2_ NP, while Co_3_O_4_ and ZnO NP had moderate effects, and CuO NP were toxic below 100 μg/mL. Nevertheless, toxicity effects of Mn_3_O_4_ and Sb_2_O_3_ NP remarkably increased over time, up to nine days. Overall, the sensitivity of the cell lines to the tested NP was comparable considering the viability data, as assessed by the resazurin assay. However, transepithelial electrical resistance (TEER) measurements showed that Caco-2 cells were more susceptible to the toxic effects of the tested NP than A549 cells.

Most of the available *in vitro* studies addressing the pulmonary toxicity of ENM were performed under submerged conditions, i.e., cultured cells are immersed in liquid media [32,33]. However, innovative approaches using advanced exposure systems that more accurately replicate the physiological aspects of the airway exposure to airborne particles and more precisely control dose deposition have emerged over the last few years [34,35]. Cellular models cultured under air-liquid interface (ALI) conditions, where aerosolised particles are directly delivered onto the cells’ surface, are regarded as a more realistic and relevant exposure system, offering a valuable alternative to the traditional submerged cultures [33,36], although most of the *in vitro* toxicology laboratories worldwide are not equipped to conduct these studies as dedicated equipment and aerosol technology is needed. Notwithstanding, several studies to assess the pulmonary toxicity of ENM under submerged and ALI conditions have been already conducted and showed that ENM hazard might be different depending on the exposure conditions [37,38,39,40].

In the present study, we comparatively investigated the *in vitro* toxicity of occupationally relevant doses of four engineered nanoparticles (ENP) used for advanced ceramics manufacture (SnO_2_, ATO, CeO_2_ and ZrO_2_ NP) in human alveolar epithelial (A549) cells under submerged vs. ALI conditions. We hypothesised that the tested ENP would be more hazardous to alveolar epithelial cells under ALI conditions compared to cells exposed under submerged conditions. To assess *in vitro* toxicity, plasma membrane integrity, cell metabolic activity (WST-1 reduction), primary and oxidative DNA damage were evaluated after exposure to the test ENP.

## 2. Materials and Methods

### 2.1. Chemicals

All chemicals used were of high purity or analytical grade. Dimethyl sulfoxide (DMSO), sodium hydroxide (NaOH), sodium chloride (NaCl), potassium chloride (KCl) and potassium hydroxide (KOH) were purchased from Merck KGaA (Darmstadt, Germany). Triton X-100, bovine serum albumin (BSA), low melting point (LMP) agarose, Tris hydrochloride (Tris-HCl), 4-(2-hydroxyethyl)piperazine-1-ethanesulfonic acid (HEPES), methyl methanesulfonate (MMS) and water TraceSELECT™ Ultra were bought from Sigma-Aldrich (Madrid, Spain). Tris-base and disodium salt dihydrate (Na_2_EDTA) were supplied from Merck Millipore (Madrid, Spain). Normal melting point (NMP) agarose was purchased from Bioline (London, UK). Potassium bromate (KBrO_3_) was supplied from Alfa Aesar (Karlsruhe, Germany). Formamidopyrimidine-DNA glycosylase (FPG) was purchased from New England Biolabs (Ipswich, MA, USA). Invitrogen™ SYBR^®^ Gold dye and CM-H_2_DCFDA (General Oxidative Stress Indicator) were bought from Thermo Fisher Scientific (Madrid, Spain). All cell culture reagents were purchased from Gibco, Thermo Fisher Scientific (Madrid, Spain).

### 2.2. Nanoparticle’s Suspensions, Aerosols Generation and Characterisation

All NP were commercial products and obtained from different suppliers in the liquid form: superlite grade SnO_2_ (10% *w*/*v*; Keeling and Walker, Stoke-on-Trent, UK), Sb_2_O_3_•SnO_2_ (ATO; 10% *w*/*v*; Keeling and Walker, Stoke-on-Trent, UK), CeO_2_ (5% *w*/*v*; PlasmaChem GmbH, Berlin, Germany) and ZrO_2_ (10% *w*/*v*; Sigma-Aldrich, Madrid, Spain). All NP suspensions under study were subjected to gamma-ray irradiation to ensure the required sterility for *in vitro* toxicity testing.

Hydrodynamic size and concentration (number of particles/mL) of the aqueous ENP suspensions under study were determined by Dynamic Light Scattering using a ZetaSizer Ultra (Malvern Panalytical, Malvern, UK) and Nanoparticle Tracking Analysis (NTA) using a NanoSight LM20 (NANOSIGHT Ltd., Salisbury, UK), respectively. The effective density of ENP suspensions was determined by measuring the pellet volume of the ENP stock suspensions after centrifugation at 2000× *g* for 2 h at 20 °C. In addition, the ENP oxidative potential (acellular ROS production) was determined by Electron Spin Resonance (ESR) based on the trapping of NP-induced hydroxyl radicals (OH) generated in the presence of hydrogen peroxide (H_2_O_2_) using DMPO (5,5-dimethyl-1-pyrroline-N-oxide) as spin trap, as previously described [39]. Briefly, NP suspensions were mixed with 0.5 M H_2_O_2_ and 0.05 M DMPO, followed by incubation for 15 min at 37 °C in a heated shaking water bath prior to ESR (MS400, Magnettech GmbH, Berlin, Germany) analysis. The ESR quantification was conducted with the Analysis Software (2.0 or higher, Magnettech GmbH, Berlin, Germany) on first derivation of ESR signals of DMPOeOH quartet as the average of total amplitudes and expressed in arbitrary units (A.U.) per sampled volume.

**Figure 1:**
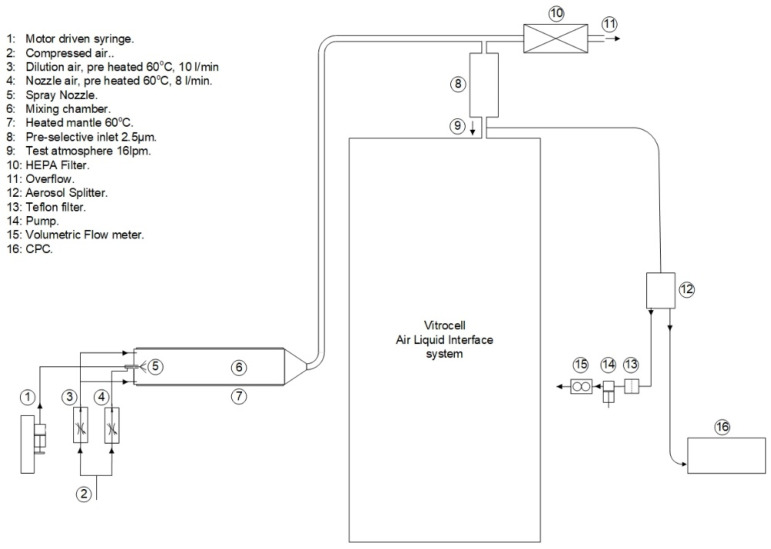
Aerosol generation set-up. Engineered nanoparticles (ENP) aerosols were generated by controlled injection of the ENP aqueous suspensions by means of a syringe pump to a spray nozzle were the liquid was nebulised using pre-heated compressed air. This aerosol was further dried and mixed in a nebulising cylinder connected to the Vitrocell^®^ automated exposure station (AES). Just before entering the AES, a Teflon filter and a condensation particle counter (CPC) were connected for aerosol characterisation.

ENP aerosols were generated as previously described [41], with minor modifications. Briefly, the ENP aqueous suspensions were fed by a syringe pump to a spray nozzle (Schlick spray-nozzle) were the liquid was nebulized using pre-heated compressed air as depicted in Figure 1. This aerosol was further dried and mixed in a nebulising cylinder. This setup was connected to an automated exposurestation (VitroCell Systems GmbH, Waldkirch, Germany) through a copper tube. Gravimetric mass concentration was determined by weighing the deposited particle mass in Teflon filters using a microbalance under controlled relative humidity (40–70%) and temperature (21–23 °C) conditions. For that purpose, the Teflon filters were weighted before and after the exposure. In addition, the aerosolised ENP deposited in grids placed in the exposure module were analysed by transmission electron microscopy (TEM) analysis and energy-dispersive X-ray spectroscopy (EDS), using a Tecnai F20 XTWIN (FEI Company, Eindhoven, The Netherlands) field emission, high-resolution transmission electron microscope operating at an accelerating voltage of 200 kV, equipped with Eagle 4k CCD camera and an EDX detector (Thermo Fisher Scientific, Waltham, MA, USA).

### 2.3. Cell Culture

Lung adenocarcinoma epithelial A549 cells from the American Type Culture Collection (ATCC^®^, CCL-185™) were cultured with RPMI 1640 medium with Glutamax™, 25 mM HEPES and supplemented with 10% heat-inactivated foetal bovine serum (FBS), 50 U/mL penicillin and 50 µg/mL streptomycin. Cells were maintained in a humidified atmosphere with 5% CO_2_ at 37 °C. To carry out the submerged exposure experiments, cells were seeded in 96-well (1.0 × 10^4^ cells/well) or 12-well plates (1.0 × 10^5^ cells/well) and allowed to adhere for 48 h at 37 °C, 5% CO_2_. For ALI exposure, cells were seeded onto 0.4 μm Corning^®^ Transwell^®^ polyester (PES) inserts (5 × 10^3^ cells/cm^2^) placed in 6- or 12-well plates and grown for 7 days.

### 2.4. Submerged vs. Air-Liquid Interface (ALI) Exposure

**Figure 2:**
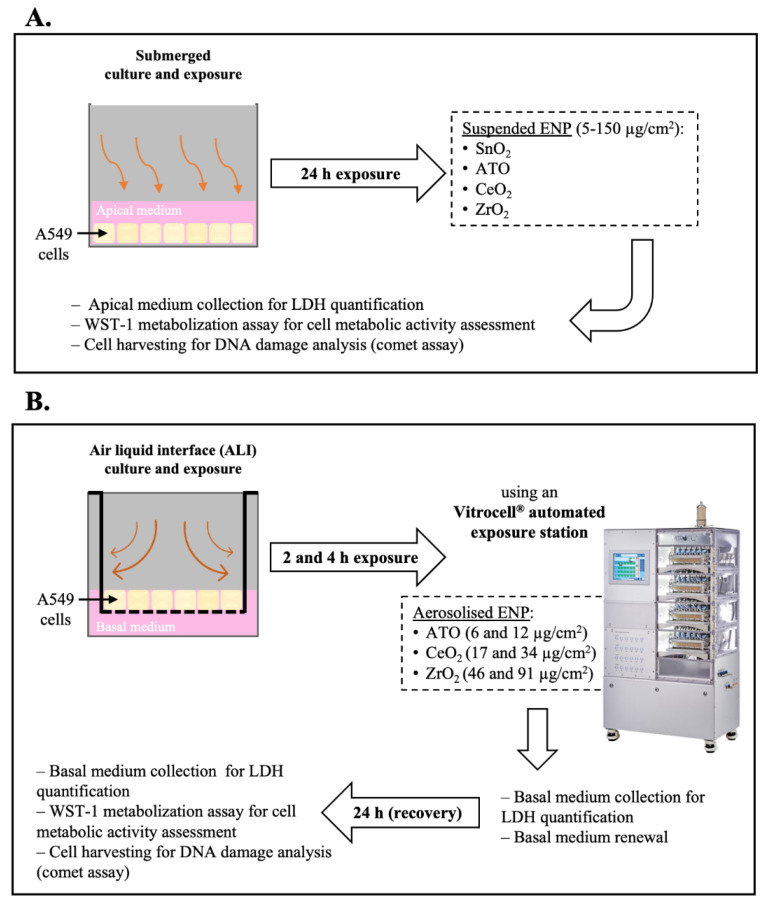
Experimental protocol scheme. (**A**) Human alveolar epithelial cultures under submerged conditions were exposed for 24 h to the tested engineered nanoparticles (ENP) dispersed in serum-free incubation medium. (**B**) Cell cultures under air-liquid interface (ALI) conditions were exposed to either clean air or ENP aerosols using an Automated Exposure Station (AES) for 2 and 4 h to achieve different deposited doses. It was not possible to generate a stable aerosol from SnO_2_ NP, though they were not tested under ALI. As depicted, samples for cytotoxicity (LDH release and WST-1 metabolisation) and genotoxicity (DNA damage) assessment were collected at different timepoints.

All NP stock suspensions under study were dispersed by indirect probe sonication using a Branson sonifier (model 450) equipped with a disruptor cup horn according with the Standard Operation Procedure (SOP) for preparation of NP suspensions developed within the NanoToxClass project (NanoToxclass, 2017). A schematic representation of the experimental protocol is depicted in Figure 2. For submerged exposure (Figure 2A), NP working concentrations were prepared from an intermediate NP suspension (300 µg/mL) by serial dilution in incubation medium (serum-free cell culture medium). Cells were immediately incubated for 24 h with the NP suspensions at 5% CO_2_ at 37 °C. For ALI exposure (Figure 2B), polarised cells grown on Transwell^®^ permeable membranes were placed inside temperature-controlled exposure modules of an automated exposure station and the cultures exposed to the NP aerosol or clean air (exposure control) at an air flow rate of 25 mL/h, under electrostatic field (1 Kv), for different timepoints (2 and 4 h) to achieve different deposited doses. The culture medium at the apical side was removed 24 h before exposure to allow cells adaptation to the ALI conditions. Cells kept in the incubator during exposure served as non-exposed controls (incubator control). Following exposure, cells were returned to the incubator, the basal compartment medium was replaced, and cells allowed to incubate for an additional 24 h (recovery time).

### 2.5. Cytotoxicity Assessment

Two endpoints were evaluated to assess the impact of the tested NP in human alveolar epithelial cells: LDH release as an indicator of plasma membrane integrity, and WST-1 reduction to evaluate the cell viability. Under submerged conditions, cells were incubated with different concentrations of the tested NP (5, 10, 25, 50, 100, 150 µg/cm^2^) and both assays carried out at 24 h after exposure. On the other hand, under ALI conditions, LDH release was assessed before exposure (to assess cell health status before exposure), immediately after exposure (basal medium from the exposure chambers was collected) and at the recovery time (24 h after exposure), while the WST-1 reduction was assessed only in the recovery time.

LDH release was determined using Roche Cytotoxicity Detection Kit (Roche, Mannheim, Germany), according to manufacturer’s instructions. Briefly, at each assessed time-point, incubation media (submerged exposure) or basolateral media (ALI exposure) were collected for analysis. Before analysis, samples from the submerged exposures were centrifuged in 96-well round bottom plates at 2210× *g* for 5 min to remove the cell debris and residual NP. Cells lysed with 2% Triton X-100 (30 min) were used as positive controls (PC). Briefly, 100 µL of freshly prepared reaction mixture was added to 100 µL of each sample and incubated up to 30 min at room temperature and protected from light. Absorbance was measured at 490 nm and 630/690 nm (reference wavelength) in a microplate reader (Spectramax M2 Molecular Devices, San Jose, CA, USA). LDH release values were normalised considering the PC mean value (total LDH release). To test for possible NP interferences with the assay, total LDH release, i.e., PC was determined in the absence and in the presence of the highest tested concentration of ENP or ENP aerosols.

Cell viability was evaluated using WST-1 Cell Proliferation Reagent Kit (Roche, Mannheim, Germany), according to the manufacturer’s instructions. For submerged samples, cells were washed with PBS pH 7.4 prior incubation with 100 µL/well of WST-1 reagent diluted 1:10 for 2 h at 37 °C, 5% CO_2_. For ALI samples, 250 µL/insert of WST-1 reagent diluted 1:10 was added to the apical compartment and let incubate for 30 min at 37 °C, 5% CO_2_. At the end of the incubation time, 100 µL were transferred to a 96-well plate. Sample’s absorbance was measured at 450 nm and 630/690 nm (reference wavelength) in a microplate reader (SpectraMax^®^ iD3 Molecular Devices, San Jose, CA, USA). WST-1 reduction values were normalised considering the control (incubator control for ALI samples) mean value.

### 2.6. Genotoxicity Assessment

Primary and oxidative DNA damage were assessed by the standard alkaline and formamidopyrimidine-DNA glycosylase (FPG)-modified comet assay versions, respectively. Cells were collected using a cell scrapper after 24 h of submerged or ALI exposure. ALI samples were suspended in cryoprotective medium (cell culture medium supplemented with 10% DMSO) and frozen at −80 °C until analysis. Cells from submerged exposures were washed 2× with PBS pH 7.4, scrapped and suspended in PBS. For submerged conditions, cells exposed to 500 µM MMS and 2.5 mM of KBrO_3_ for 30 min were included as PC of the primary and oxidative DNA damage, respectively, whereas for ALI cells exposed to 1 mM H_2_O_2_ for 30 min were used as PC. Cells were counted in a Neubauer’s chamber and 6.0 × 10^3^ cells were transferred to a microcentrifuge tube and centrifuged at 700× *g* for 5 min. Supernatant was removed and cells were resuspended in 100 μL of 1% LMP agarose. Five microliters were placed onto microscope slides precoated with 1% NMP, using a high-throughput system of 12-minigel comet assay unit (Severn Biotech Ltd.^®^, Kidderminster, UK). Three slides were prepared, one for the standard alkaline comet assay and two for the enzyme-modified version (with or without FPG-enzyme), and duplicates of each sample were added to each slide. The alkaline comet assay procedure was performed as previously described (Bessa et al., 2019). After agarose solidification at 4 °C for 5 min, slides were immersed in ice-cold lysis solution (2.5 M NaCl, 100 mM Na_2_EDTA, 10 mM Tris-base, 10 M NaOH, pH 10, 1% Triton-X 100) during 1 h at 4 °C, protected from light. After lysis, FPG-modified comet assay slides were washed three times for 5 min with buffer F (0.1 M KCl, 0.5 mM Na_2_EDTA, 40 mM HEPES, 0.2 mg/mL BSA, pH 8) prior incubation for 30 min at 37 °C with 2.7 U/mL of FPG enzyme or with buffer F alone. After incubation, FPG and buffer F slides were washed with PBS pH 7.4. The alkaline comet assay slides were washed 3 times with PBS pH 7.4 for 5 min. For DNA unwinding, all slides were immersed in electrophoresis solution (1 mM Na_2_EDTA, 0.3 M NaOH, pH 13) for 40 min at 4 °C, followed by electrophoresis in the same solution for 30 min at a constant 25 V (0.9 V/cm) and 400 mA. At the end of electrophoresis, slides were neutralised and fixed as described elsewhere [42]. For the comet scoring, slides were initially hydrated in Tris-EDTA (TE) buffer (10 mM Tris-HCl, 1 mM Na_2_EDTA, pH 7.5–8) and then stained with 1:10,000 dilution of SYBR^®^ Gold in TE buffer for 40 min at room temperature. Comets were visualised in a Motic BA410 ELITE series microscope equipped with a complete EPI-fluorescence kit and scored using the Comet Assay IV image analysis software (Perceptive Instruments, Staffordshire, UK). At least 100 cells/experimental group (50 in each replicate gel) were scored and the mean of the percentage of DNA in the comet tail (% tail intensity) was used as a DNA damage descriptor.

### 2.7. Statistical Analysis

Statistical analysis was performed using SPSS (version 26.0, Armonk, NY, USA) and GraphPad Prism (version 6.0, San Diego, CA, USA) statistical software. Experimental data were expressed as mean ± standard deviation (SD). Data were tested for normality and homogeneity of variances by Shapiro–Wilk and Levene’s tests, respectively. For each assessed timepoint, differences between tested doses and controls were estimated using a one-way analysis of variance (ANOVA) followed by post-hoc Dunnett’s test for multiple comparisons. A *p* value < 0.05 was considered significant.

## 3. Results

### 3.1. Nanoparticle’s Suspensions and Aerosols Characterisation

In Table 1 are presented the main physicochemical features of the tested ENP suspensions. As shown, mean particle sizes of 455.5 nm, 688.5 nm, 305.6 nm and 406.0 nm were obtained for SnO_2_, ATO, CeO_2_ and ZrO_2_ NP, respectively. A slight increase compared to the negative control but no significant differences in the oxidative potential of the four tested ENP were detected suggesting that all tested particles have a low ability to produce •OH in a cell-free environment.

Under submerged conditions, all ENP are expected to settle onto the cells after 24 h of exposure since ENP effective density is substantially higher compared to cell culture medium. Regarding ALI exposure, it was not possible with the limited available amount of test material to generate a stable aerosol from the SnO_2_ NP, thus this NP was not tested under these conditions. Table 2 shows NP aerosolisation conditions and aerosol deposition in human alveolar epithelial cultures. The deposited doses were calculated from the gravimetric data. Average single doses ranged between 6 to 12 μg/cm^2^ for ATO NP, 46 to 92 μg/cm^2^ for CeO_2_ NP, 17 to 34 μg/cm^2^ for ZrO_2_ NP.

**Figure 3:**
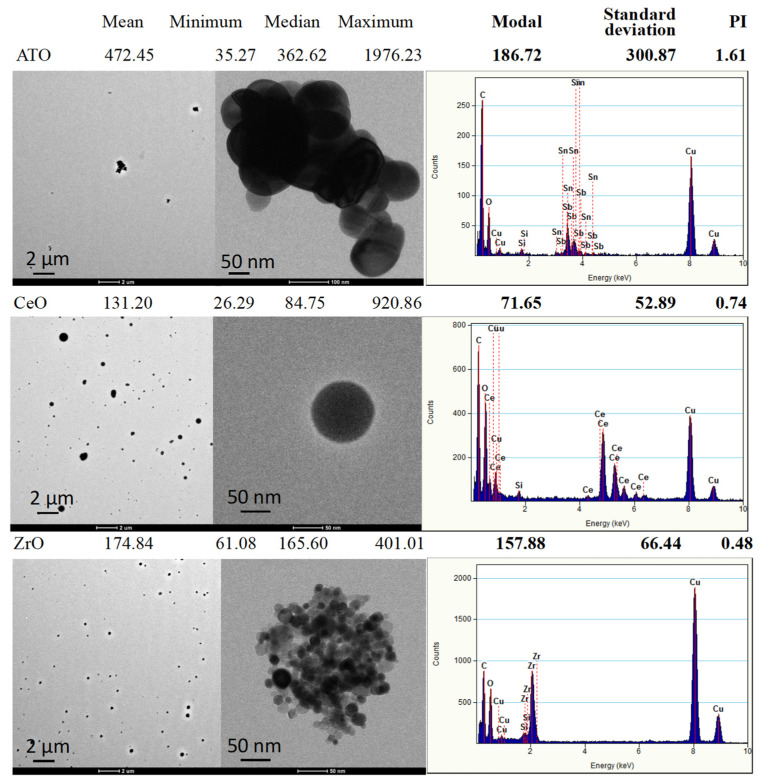
Representative transmission electron microscopy (TEM) images of the generated aerosols (EDS spectra) with respective size distribution values. The size distribution of aerosol generated particles was determined from TEM images by using the ImageJ software.

Analysis of the generated aerosols collected on TEM grids (Figure 3) showed that NP exhibited different shapes and size distributions. The ATO aerosolised sample is composed of larger, irregular agglomerations (up to 2 μm) of fused small spheroidal NP (50–100 nm) with mean particle sizes of 472.45 nm and a modal value (value with maximum count) of 186.72 nm that give rise to a calculated PI polydispersity index) of 1.61. CeO_2_ aerosols present themselves as spherical but with broad distribution NP (from 26 to 920 nm) with a mean value of 131.2 nm and a modal value of 71.65 nm associated with a PI of 0.74. ZrO_2_ aerosols are formed of apparently spherical agglomerations (up to 400 nm) of very small round NP (10–25 nm) giving a mean value of the agglomerations of 174.8 nm, a modal value of 157.88 nm with a PI of 0.48.

### 3.2. Cytotoxicity: Submerged vs. ALI Conditions

**Figure 4:**
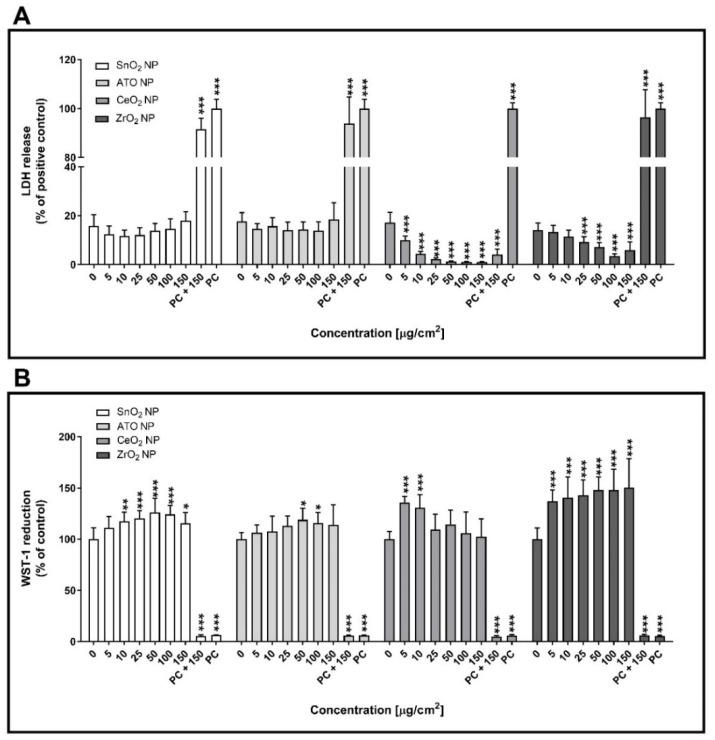
Cytotoxicity of the tested engineered nanoparticles (ENP) (SnO_2_, ATO, CeO_2_ and ZrO_2_) in human alveolar epithelial cells under submerged conditions being exposed for 24 h. Lactate dehydrogenase release (LDH) release (**A**) and WST-1 reduction (**B**) assays were carried out after 24 h exposure to the NP suspensions prepared in serum-free cell culture medium. Data are expressed as mean ± standard deviation (*n* = 3–4). LDH release values were normalised considering the positive control (total LDH release; cells lysed with 2% Triton X-100), while WST-1 reduction values were normalised considering the negative control. Data was analysed by the one-way analysis of variance (ANOVA) test followed by the Dunnett’s post hoc test for multiple comparisons. * *p* ≤ 0.05, ** *p* ≤ 0.01 and *** *p* ≤ 0.001 vs. negative control. PC: Positive control.

Figure 4 shows the cytotoxicity data for the SnO_2_, ATO, CeO_2_ and ZrO_2_ NP under study, as assessed by the LDH release and WST-1 viability assays. As depicted, no significant changes in plasma membrane integrity of human alveolar epithelial cells exposed to SnO_2_ or ATO NP compared to control cells were observed under submerged conditions at 24 h after exposure (Figure 4A). On the other hand, a clear concentration-dependent decrease in LDH release was observed in cells exposed to CeO_2_ or ZrO_2_ NP compared to the negative controls (*p* ≤ 0.001). However, CeO_2_ NP seem to interfere in the LDH assay, as total LDH release of the cells exposed to the highest tested concentration (PC + 150; 4.08 ± 2.23%) was far below the total LDH release in the absence of CeO_2_ NP (PC; 100.00 ± 2.37%). This finding is most likely caused by CeO_2_ NP deposition onto the cell monolayer preventing LDH leakage into the extracellular environment. Regarding cellular viability, significant increases in WST-1 reduction were observed in cells exposed to all tested NP at 24 h exposure (*p* ≤ 0.001) (Figure 4B). Taken together, these results seem to indicate that all tested NP did not induce significant cytotoxic responses in human alveolar epithelial cells under submerged conditions.

**Figure 5:**
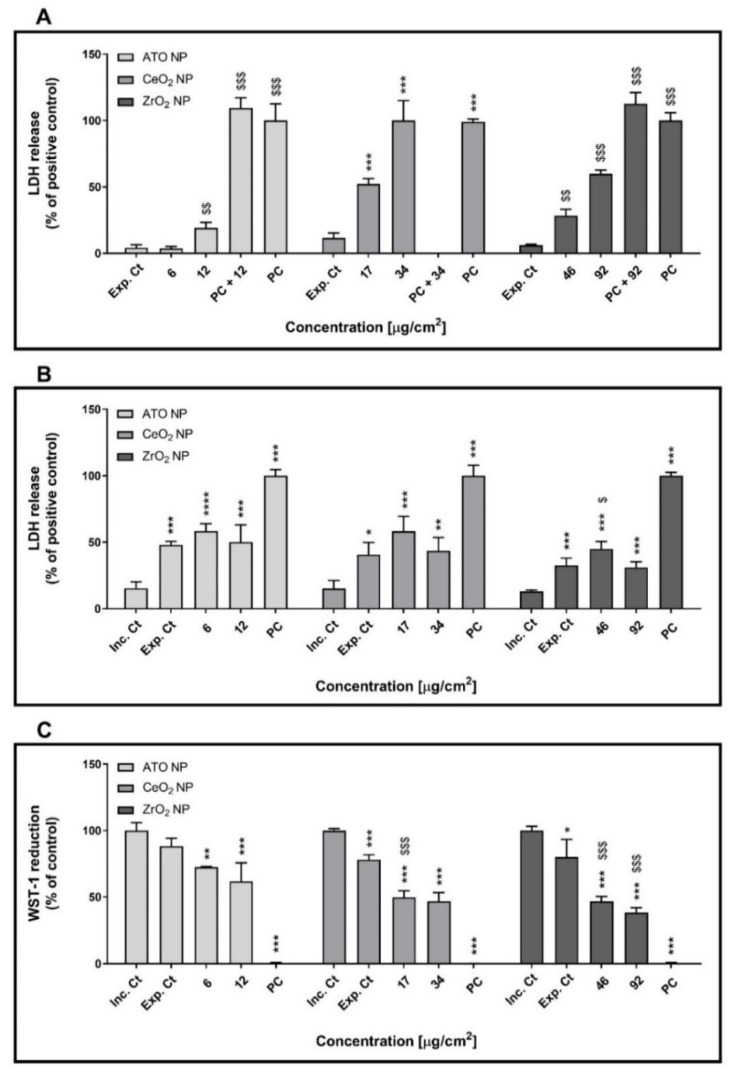
Cytotoxicity of the aerosolised engineered nanoparticles (ENP) (ATO, CeO_2_ and ZrO_2_) in polarised cultures of human alveolar epithelial cells at air-liquid interface (ALI) conditions. Lactate dehydrogenase release (LDH) was assessed immediately after (0 h) (**A**) and at 24 h (**B**) after exposure. (**C**) WST-1 reduction assay was carried out only in the recovery period (24 h after exposure). Data are expressed as mean ± standard deviation (*n* = 3). LDH values were normalised considering positive control (total LDH release; cells lysed with 2% Triton X-100), while WST-1 values were normalised considering the incubator control. Data was analysed by the one-way analysis of variance (ANOVA) test followed by the Dunnett’s post hoc test for multiple comparisons. * *p* ≤ 0.05, ** *p* ≤ 0.01 and *** *p* ≤ 0.001 vs. Inc. Ct; ^$^ *p* ≤ 0.05, ^$$^ *p* ≤ 0.01 and ^$$$^ *p* ≤ 0.001 vs. Exp. Ct. Inc. Ct: Incubator control; Exp. Ct: Exposure control; Positive Ct: Positive control.

Figure 5 refers to the cytotoxicity of the aerosolised ATO, CeO_2_ and ZrO_2_ NP in human alveolar epithelial cells at ALI. As expected, before exposure, no effects on the LDH release were observed in control, an indicator of cell health (data not shown). Immediately after exposure to all the tested aerosolised NP, a significant increase in LDH release was observed compared to cells exposed to clean air (exposure control). This detrimental effect on plasma membrane integrity was more marked in cells exposed to the highest deposited dose of CeO_2_ (34 µg/cm^2^; 52.36 ± 3.15%) and ZrO_2_ (92 µg/cm^2^; 59.77 ± 2.46%) NP aerosols than to ATO NP (12 µg/cm^2^; 19.11 ± 3.43%) (Figure 5A). Based on LDH release data, calculated half-maximal effective concentrations (EC_50_) were of 74.77 (CI 95%: 66.51–84.05), 32.97 (CI 95%: 31.01–35.04) and 20.70 (CI 95%: 12.60–33.98) μg/cm^2^ for ATO, CeO_2_ and ZrO_2_ NP respectively. Nevertheless, at 24 h after exposure, no differences in LDH release levels were observed among the exposed cells (i.e., exposure control and NP aerosol-exposed cells), although those were significantly higher than the incubator control (Figure 5B). However, a significant decrease in cellular metabolic activity of similar magnitude, as assessed by the WST-1 assay, was observed at 24 h after exposure to all tested aerosolised NP (Figure 5C).

### 3.3. Genotoxicity: Submerged vs. ALI Conditions

**Figure 6:**
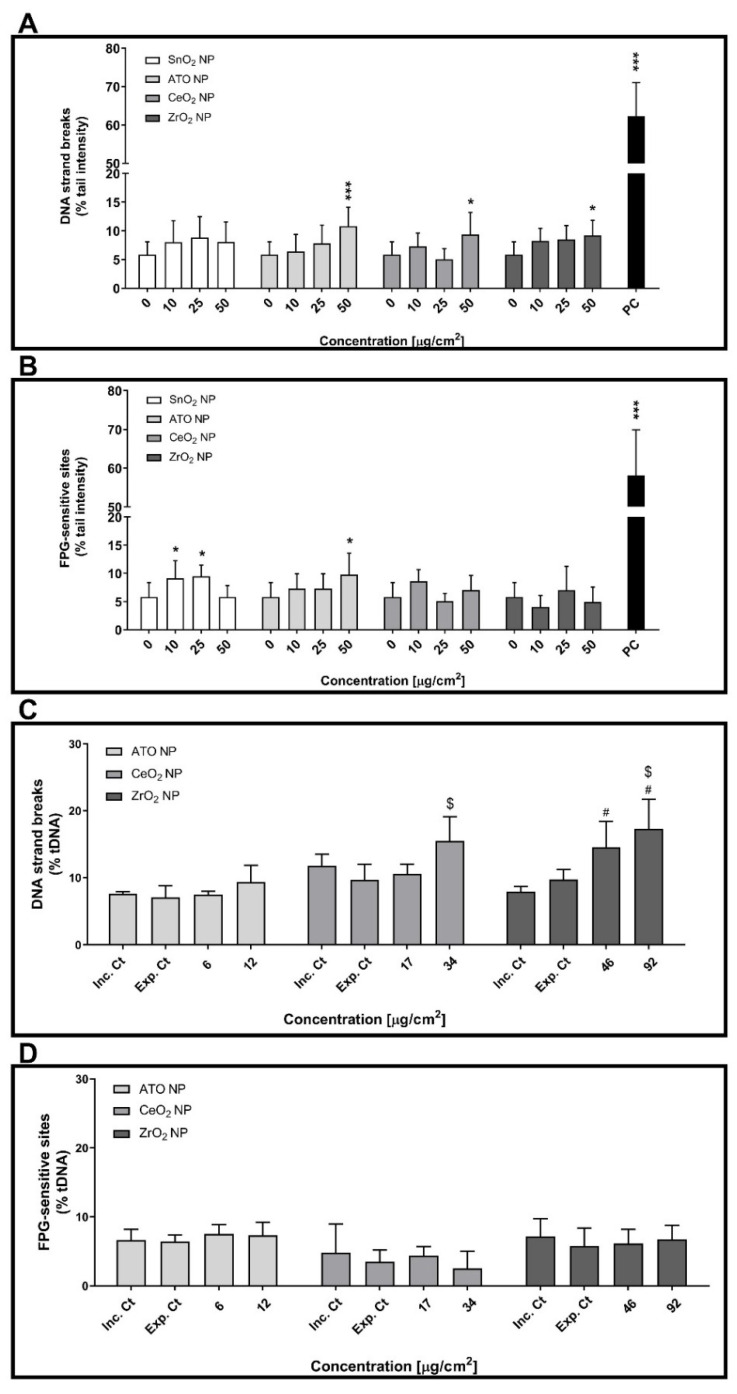
Genotoxicity of the tested engineered nanoparticles (ENP) in human alveolar epithelial cells under submerged (**A**,**B**) and ALI (**C**,**D**) conditions. Primary (**A**,**C**) and oxidative (**B**,**D**) DNA damage were assessed at 24 h after exposure to the ENP suspensions by the alkaline and FPG-modified comet assay versions, respectively. Data are expressed as mean ± standard deviation (*n* = 3–4). Data was analysed by one-way ANOVA followed by Dunnett’s post-hoc test. * *p* ≤ 0.05 and *** *p* ≤ 0.001 vs. negative control. ^#^ *p* ≤ 0.05 vs. incubator control and ^$^ *p* ≤ 0.05 vs. exposure control. PC: Positive control; 500 µM MMS and 2.5 mM KBrO_3_ for primary (**A**) and oxidative (**B**) DNA damage under submerged conditions, respectively.

The comet assay was performed to assess the primary (strand breaks) and oxidative (FPG-sensitive sites) DNA damage levels of cells exposed to suspended or aerosolised NP (Figure 6). For cells cultured under submerged conditions, three non-cytotoxic concentrations of ATO, CeO_2_ and ZrO_2_ NP were tested: 10, 25 and 50 µg/cm^2^. At 24 h post-exposure, increased levels of DNA strand breaks were observed in cells incubated with the highest concentration (50 µg/cm^2^) of any tested NP compared to control cells (Figure 6A). On the other hand, cells exposed to SnO_2_ and ATO NP but not to CeO_2_ and ZrO_2_ NP exhibited a significant increase of DNA oxidative lesions compared to control cells (Figure 6B). While cells exposed to 10 or 25 µg/cm^2^ of SnO_2_ NP (9.14 ± 3.11 and 9.47 ± 2.00% tDNA, respectively) showed increased levels of FPG-sensitive sites, only cells exposed to the highest tested concentration of ATO NP (50 µg/cm^2^; 9.77 ± 3.79% tDNA) exhibited increased levels of DNA oxidative lesions compared to control cells (5.80 ± 2.60% tDNA). As expected, high levels of primary and oxidative DNA damage were detected for submerged cells exposed to the corresponding PC (MMS 500 µM: 62.23 ± 8.85% tDNA; KBrO_3_ 2.5 mM: 58.16 ± 11.73% tDNA, respectively).

The data obtained for human alveolar epithelial cells exposed to the NP aerosols at ALI is depicted in Figure 6C,D. As shown, exposure to aerosolised ATO NP failed to affect DNA integrity. However, cells exposed to the highest deposited dose of CeO_2_ NP aerosols exhibited increased levels of DNA strand breaks (34 µg/cm^2^; 15.48 ± 3.64% tDNA) (Figure 6C). Regarding ZrO_2_ NP, a concentration-dependent increase of DNA strand breaks was detected in cells exposed to these aerosols compared to control cultures (Figure 6C). Notwithstanding this, no significant changes in oxidative DNA damage were detected for all the tested NP aerosols (Figure 6D).

**Figure 7:**
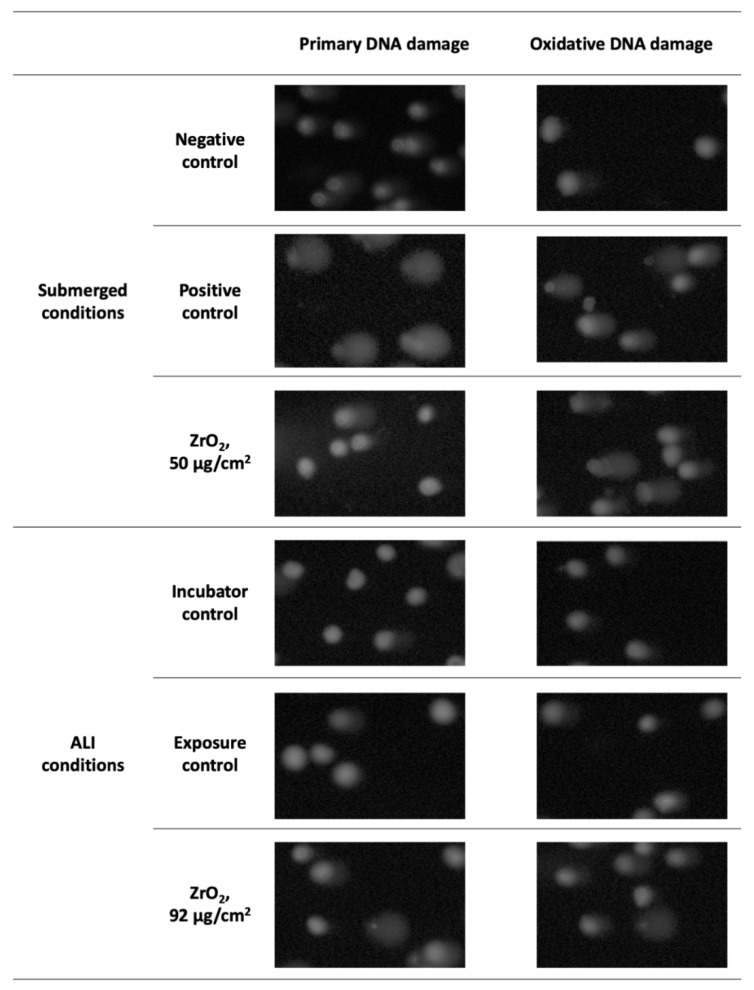
Comet assay representative images (100× magnification) of human alveolar epithelial cells under submerged and ALI conditions exposed to the highest tested concentration of ZrO_2_ NP and respective experimental controls.

Representative comet images of human alveolar epithelial cells exposed to ZrO_2_ NP, which were able to induce DNA damage both under submerged and ALI conditions are depicted Figure 7.

As shown, wider comet tails were observed in cells exposed to the highest concentration of ZrO_2_ NP, either in submerged or ALI conditions, when compared to those obtained in the negative controls. A pronounced DNA damage in relation to control was observed in cells at ALI exposed to the PC (1 mM H_2_O_2_, 30 min), which could not be quantified using the comet image analysis software (data not shown).

## 4. Discussion

Herein, we have comparatively evaluated the *in vitro* toxicity of four industrially relevant ENP in human alveolar epithelial-like submerged cultures exposed to liquid suspensions or in ALI cultures exposed to aerosolised ENP. Although not exactly the same, the tested dose levels were comparable as they were within the same range: 5–150 µg/cm^2^ for submerged cultures and 6–92 µg/cm^2^ for ALI cultures. From a human exposure scenario point of view, these values are relevant considering that the estimated lifetime dose under realistic ambient conditions is 6.6 µg/cm^2^, while for a worst-case occupational exposure scenario a daily alveolar mass dose of 0.13 µg/cm^2^ and a maximum accumulated lifetime dose of 420 µg/cm^2^ are expected to be achieved [36].

Overall, our data showed that ENP cytotoxicity in human alveolar epithelial cells was more evident under ALI than at submerged conditions. Under ALI conditions, based on the EC_50_ values for LDH release immediately after exposure, ENP can be ranked for their toxicity hazard as follows: ZrO_2_ NP > CeO_2_ NP > ATO NP. Interestingly, no significant differences in the LDH release at 24 h post-exposure (recovery time) between cells exposed to clean air (exposure control) and cells exposed to the ENP aerosols were detected. However, a slight increase in LDH release in exposure control cells was detected compared to the incubator control, suggesting that plasma membrane integrity might have been affected by the air flow across the cells, considering the lack of tight intercellular junctions that polarised A549 cells exhibit [28,41]. Accordingly, other respiratory cell models such as bronchial epithelial Calu-3 cells have been shown to be more suitable for continuous flow exposure systems such as the one employed in the present study [41,43]. Notwithstanding, a significant decrease in cellular metabolic activity of cells exposed to ENP aerosols compared to the exposure control has been detected at 24 h post-exposure, meaning that the aerosolised ENP negatively affected the cell physiology.

In submerged conditions, no significant cytotoxic effects were observed in human alveolar epithelial cells exposed to the liquid suspensions of ENP. This difference in the cytotoxic potential of the tested ENP in submerged vs. ALI exposure conditions may obviously arise from differences in the attained deposited doses in both exposure conditions. One important aspect that also differed between exposure conditions is the potential for NP interference in the LDH release assay, in particular for CeO_2_ NP that clearly affected the assay as evidenced by the low levels of LDH release comparing with the control and the evident difference in the PC value that corresponds to the maximum release of LDH, in the absence and in the presence of CeO_2_ NP.

Regarding the genotoxic potential of the tested ENP, our data showed that all tested ENP seem to increase the primary DNA damage of human alveolar epithelial cells regardless of the exposure mode, except for ATO NP, where cells exposed in ALI conditions did not show significant changes in the level of DNA strand breaks comparing with the controls. Moreover, human alveolar epithelial cells seem to be more sensitive to the genotoxic effects of ZrO_2_ NP aerosols than to the same NP in liquid medium. Nonetheless, as stated above, this apparent difference in sensitivity to the tested ZrO_2_ NP might be related with differences in the physicochemical features and/or deposited doses under the two exposure conditions. However, while SnO_2_ and ATO NP caused DNA oxidative lesions in cells under submerged cultures, no changes in FPG-sensitive sites were detected at ALI exposure.

Our data are in line with previous reports on *in vitro* toxicity of the tested ENP in human alveolar epithelial-like A549 cells under submerged conditions. Tabei, et al. [44] have reported low levels of NP uptake and no evident cytotoxic effects in A549 cells exposed for 6 and 24 h to indium-doped SnO_2_ NP (30 nm; 1–1000 μg/mL), in spite of a markedly increase in ROS levels, expression of heme oxygenase 1 (HO-1) gene and DNA damage have been observed [44]. Titma, Shimmo, Siigur and Kahru [31] also reported no significant cytotoxicity in A549 cells exposed for 24 h to 3–100 µg/mL of Sb_2_O_3_ NP, though a marked increase in toxicity has been observed after long-term exposure (up to 9 days) [31]. Regarding CeO_2_ NP, some studies in the literature showed that these NP are relatively non-cytotoxic. Indeed, minimal or no effects on cell viability and LDH release were detected in A549 alveolar epithelial cells exposed to CeO_2_ NP in liquid incubation medium (concentrations up to 100 µg/mL [45,46,47] and 1000 µg/mL [48]), although some authors observed induction of genotoxicity (DNA damage; 0.5 μg/mL to 5000 μg/mL) [49]. On the other hand, some studies demonstrated that CeO_2_ NP induced plausible toxicity effects towards A549 cells. For instance, Mittal and Pandey [50] suggested that CeO_2_ NP produced an increased amount of ROS, which majorly contributed to extensive DNA damage and cell cycle arrest, responsible for apoptotic cell death in A549 cells [50]. According to these authors, CeO_2_ NP induced a concentration-dependent increase in ROS production up to 6 h, however this tendency was strongly attenuated after 24 h exposure [50], in opposition to what was found in the present study. Lanone, Rogerieux, Geys, Dupont, Maillot-Marechal, Boczkowski, Lacroix and Hoet [30] assessed the toxicity of CeO_2_ and ZrO_2_ (0–5000 µg/mL) in the human alveolar epithelial A549 and macrophage THP-1 cell lines at 24 h after exposure and found that both CeO_2_ and ZrO_2_ NP caused moderate cytotoxicity [30]. Recently, our lab has observed a mild cytotoxicity after exposure to aerosolised ATO and ZrO_2_ NP at early timepoints (24 h; 5.56 µg ATO/cm^2^ and 10.98 µg ZrO_2_/cm^2^) but no significant changes for late timepoints (72 h) in human 3D cultures of bronchial epithelial MucilAir™ cultures, with no meaningful effects regarding DNA damage [51].

Our data support the view that the ENP are more toxic to human alveolar epithelial cells when aerosolised rather than applied as a liquid suspension in submerged cell cultures. Lenz, et al. [38] compared the oxidative stress and proinflammatory responses of A549 exposed to aerosolised zinc oxide (ZnO) nanoparticles under ALI and submerged conditions. Lower levels of proinflammatory markers (IL-8, IL-6, and GM-CSF) were found in cells exposed under ALI conditions compared to submerged cultures, accompanied by no significant effects on the transcript levels of oxidative stress markers (0.7 and 2.5 μg ZnO/cm^2^) [38]. Panas, et al. have also compared the biological responses of A549 cells under ALI or submerged cultures after exposure to two types of amorphous SiO_2_ NP [40]. Amorphous SiO_2_ NP induced similar cellular responses in both cultures systems, although submerged exposure to SiO_2_ NP triggers stronger effects at much lower cellular doses [40]. On the other hand, Medina-Reyes, et al. [33] investigated the biological responses in A549 cells exposed to TiO_2_ nanofibers and NP. These authors found that cytotoxicity of TiO_2_ nanofibers and NP was similar in both types of A549 culture, although their uptake was higher in submerged compared to ALI cultures. TiO_2_ nanofibers induced higher DNA double strand breaks (DSB) in A549 cells under ALI conditions than in submerged cultures, though TiO_2_ NP caused similar levels of DSB in both culture conditions [39]. Recently, Diabaté, et al. [48] evaluated the *in vitro* toxicity of CeO_2_ and TiO_2_ NP in monocultures of A549 cultured at ALI vs. co-cultures of A549 and THP-1 macrophages under submerged conditions. Similar to our study, cells under ALI conditions were more sensitive to NP-induced toxicity when compared to those cultured under submerged conditions. Moreover, CeO_2_ NP induced moderate *in vitro* toxicity, whilst TiO_2_ NP caused evident cytotoxicity, pro-inflammatory gene expression and genotoxicity [52]. Taken together, these studies suggest that cell response to NM is dependent upon the exposure conditions that includes sample preparation but also upon the physicochemical properties of the NM. It is important to point out that *in vitro* pulmonary models in submerged systems do not fully recapitulate relevant cellular and physiological airway epithelia features [33,36]. In vivo, airways are not fully covered by pulmonary fluid to allow the gas-exchange between cells and the environment. Indeed, exposure to inhaled toxicants such as airborne NP mainly occurs under ALI conditions [53]. Thus, *in vitro* exposure systems able to deliver aerosolised particles to cells cultured at ALI is of major importance for a more reliable *in vitro* testing of NP effects in pulmonary nanotoxicity studies, and more accurately mimicking the human in vivo cells in the respiratory tract rather than the conventional approaches using *in vitro* submerged cell cultures [25,54].

More pronounced cytotoxic effects were observed after exposure to the aerosolised NP, while a similar DNA damage after NP exposure was found for both types of exposure conditions (except for ATO NP). The observed differences in toxicity may arise from different deposited doses attained in the cell surface when covered in culture medium or air, which consequently influences the toxic potency of these NP, as well as their capacity to interfere with the assay components. The dose levels tested herein are within the lifetime dose under realistic occupational exposure to NP, and the results obtained reflect the negative impact these aerosolised nano-sized materials inadvertently have on the workers’ health. Although both submerged and ALI cell culture systems enable the evaluation of NP toxicity *in vitro*, the present study highlights how realistic dose levels under ALI conditions provide more biologically valuable data regarding occupational exposure to airborne NP.

So far, it has been difficult to assert with certainty whether airborne ENP constitute a higher or lower hazard to humans compared to incidental, process-generated NP since there are few toxicity studies on the latter. We have recently showed that both fine and NP fractions released and collected during high-velocity oxy-fuel (HVOF) spraying at an industrial facility induced higher toxicity than two ENP (ATO and ZrO_2_ NP) on bronchial epithelial MucilAir^TM^ cultures under ALI conditions, most likely due to their chemical complexity [51]. These findings emphasize the importance of investigating not only ENP but also incidental, process-generated NP hazards, to have a deeper understanding of the toxicity mechanisms and potential risks for workers’ health from occupational exposure to these NP.

## 5. Conclusions

Different toxicity effects induced by ENP used as raw materials in the advanced ceramics industry were observed in human alveolar epithelial cells under both types of culture condition. As hypothesised, ENP seemed more hazardous to human alveolar epithelial cells cultured under ALI compared to submerged conditions. ALI cultures are a key strategy for future occupational inhalation NP toxicity studies as it also has more potential to extrapolate the finding for human risk assessment. Additionally, from an occupational health management point of view, the study of the toxicity in different exposure systems is of utmost importance to better assess the potential impact on workers’ health of a material in various exposure scenarios, to identify their hazards and put them in their true perspective.

## Figures and Tables

**Table 1:** Physicochemical characteristics of the tested engineered nanoparticles (ENP) stock suspensions.

ENP	Hydrodynamic Size (nm)	Concentration (Number of Particles/mL)	Oxidative Potential (A.U.) *	Effective Density (mg/mL)
SnO_2_	455.5 ± 17.98	2.70 × 10^8^	4958	6.7
ATO	688.5 ± 97.80	12.28 × 10^8^	4081	17.4
CeO_2_	305.6 ± 79.72	8.07 × 10^8^	4806	1.5
ZrO_2_	406.0 ± 1.79	22.05 × 10^8^	3408	3.5

Data are presented as mean ± SD. Hydrodynamic size was measured by Dynamic Light Scattering (DLS). Concentration was determined by Nanoparticle Tracking Analysis (NTA). Oxidative potential was measured by Electronic Spin Resonance (ERS). A.U.: arbitrary units. * Negative control (ultrapure water) = 3191 A.U.; Positive control (DOFA) = 48,041 A.U.

**Table 2:** Aerosolisation conditions and exposure concentrations of the tested aerosolised engineered nanoparticles in human alveolar epithelial-like cultures.

		ATO	CeO_2_	ZrO_2_
Liquid suspension flow rate (mL/h)		0.6	1.2	0.6
Aerosol flow through the insert (mL/min)		25	25	25
Aerosol concentration (mg/m^3^)		2.3	6.4	17.0
Number of particles		4 × 10^5^	1 × 10^5^	1 × 10^5^
Deposited mass	2 h	6	46	17
	4 h	12	92	34

Aerosol mass concentration determined by gravimetry; Number of particles determined using a condensation particle counter (CPC); Deposited mass = mass concentration of aerosol/volume of aerosol passing through exposure chambers during exposure.
